# The genome sequence of the Chalkhill Blue, *Lysandra coridon* (Poda, 1761)

**DOI:** 10.12688/wellcomeopenres.19253.1

**Published:** 2023-04-12

**Authors:** Roger Vila, Konrad Lohse, Alex Hayward, Dominik R. Laetsch, Charlotte Wright, Wellcome Sanger Institute Tree of Life programme, Wellcome Sanger Institute Scientific Operations: DNA Pipelines collective, Tree of Life Core Informatics collective, Darwin Tree of Life Consortium

**Affiliations:** 1 Institut de Biologia Evolutiva (CSIC - Universitat Pompeu Fabra), Barcelona, Spain; 2 Institute of Ecology and Evolution, University of Edinburgh, Edinburgh, Scotland, UK; 3 College of Life and Environmental Sciences, Department of Biosciences, University of Exeter, Exeter, England, UK; 4 Wellcome Sanger Institute, Hinxton, England, UK

**Keywords:** Lysandra coridon, Chalkhill Blue, genome sequence, chromosomal, Lepidoptera

## Abstract

We present a genome assembly from an individual male *Lysandra coridon* (the Chalkhill Blue; Arthropoda; Insecta; Lepidoptera; Lycaenidae). The genome sequence is 541 megabases in span. Most of the assembly is scaffolded into 90 chromosomal pseudomolecules, including the assembled Z sex chromosome. The mitochondrial genome has also been assembled and is 15.4 kilobases in length. Gene annotation of this assembly on Ensembl identified 13,334 protein coding genes.

## Species taxonomy

Eukaryota; Metazoa; Ecdysozoa; Arthropoda; Hexapoda; Insecta; Pterygota; Neoptera; Endopterygota; Lepidoptera; Glossata; Ditrysia; Papilionoidea; Lycaenidae; Polyommatinae; Polyommatini; Polyommatina; *Lysandra*; *Lysandra coridon* (Poda, 1761) (NCBI:txid268709).

## Background

The Chalkhill Blue ( *Lysandra coridon*) is a species of butterfly that typically inhabits calcareous grasslands throughout Europe. In the UK, *L. coridon* is considered vulnerable ( Fox *et al.*, 2022), however, it is listed as Least Concern in the IUCN Red List (Europe) ( Van Swaay *et al.*, 2010).

Males possess pale metallic blue upperside wings while females are usually dark brown, although female blue forms exist (f. *syngrapha*). Both sexes have chequered wing fringes and a brown margin, with brown spots encircled with white, most visible on the hindwings ( Schmitt, 2015). The underside wing of both sexes has multiple black spots with a white margin and row of submarginal orange markings, on a variable background ranging from whitish or grey to brownish. This species is sedentary, staying largely within local areas, which can reach high population density ( Asher *et al.*, 2001; Schmitt *et al.*, 2006). A single brood flies between mid-June and September. Larvae feed primarily on horse-shoe vetch *Hippocrepis comosa*, and have a myrmecophilous relationship with ants ( Fiedler & Maschwitz, 1988).

**Figure 1.**
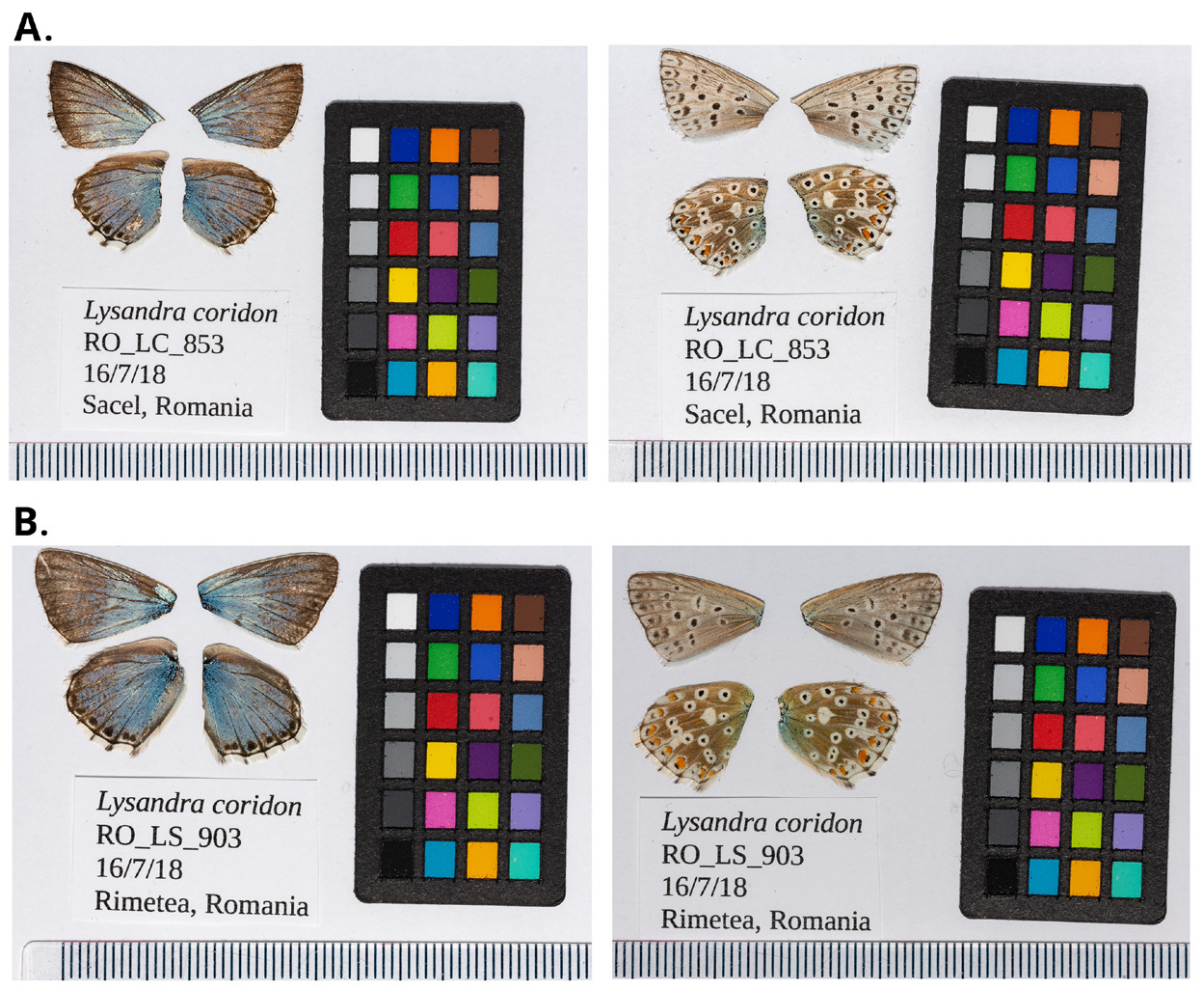
Forewings and hindwings of *Lysandra coridon* specimens from which the genome was sequenced. **A**. Dorsal (left) and ventral (right) surface view of wings from specimen RO_LC_853 (ilLysCori1) from Săcel, Cluj, Romania, used for genome sequencing. **B**. Dorsal (left) and ventral (right) surface view of wings from specimen RO_LS_903 (ilLysCori2) from Rimetea, Romania, used for Hi-C scaffolding.

Allozyme and mitochondrial gene studies have demonstrated the existence of two major genetic lineages of *L. coridon* ( Dapporto *et al.*, 2022; Schmitt & Seitz, 2001; Talavera *et al.*, 2013): a Western lineage that inhabits the UK, Spain, France, Italy, much of the Alps and most of Germany, and an Eastern lineage which is found in the Balkans, Poland, northern Germany and the rest of eastern Europe. Interestingly, *L. coridon* displays a gradient of populations with increasing chromosome number from west to east, encompassing from 87 to 93 chromosomes ( de Lesse, 1969). Here we present a chromosomally complete genome sequence for *L. coridon*, based on one male specimen from Săcel, Cluj, Romania ( Figure 1).

### Genome sequence report

The genome was sequenced from one male *L. coridon* specimen collected from Săcel, Cluj, Romania (latitude 46.61, longitude 23.46). A total of 47-fold coverage in Pacific Biosciences single-molecule HiFi long reads and 60-fold coverage in 10X Genomics read clouds was generated. Primary assembly contigs were scaffolded with chromosome conformation Hi-C data. Manual assembly curation corrected 242 missing or mis-joins and removed 10 haplotypic duplications, reducing the scaffold number by 62.21% and increasing the scaffold N50 by 91.5%.

**Figure 2.**
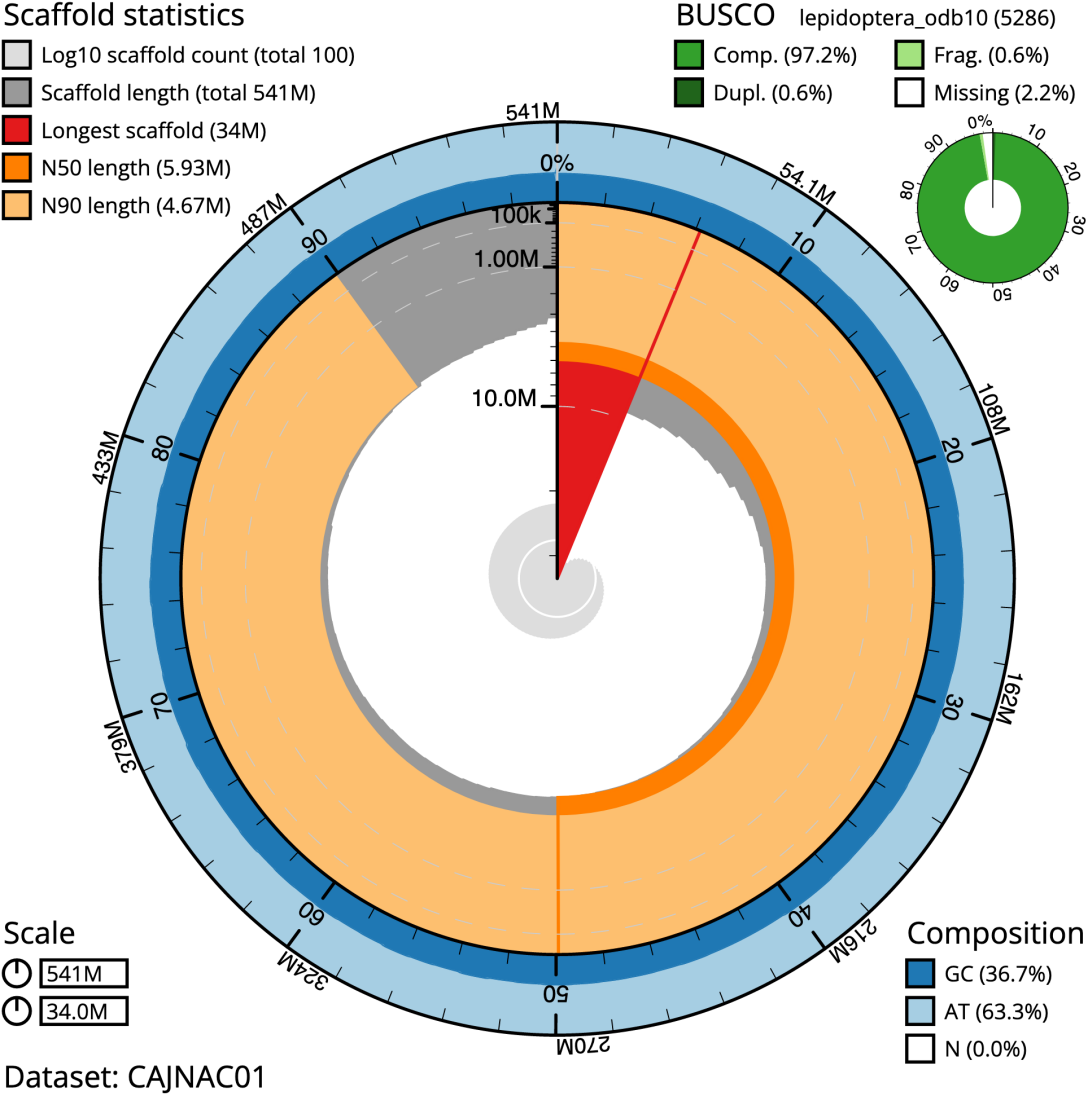
Genome assembly of *Lysandra coridon*, ilLysCori1.1: metrics. The BlobToolKit Snailplot shows N50 metrics and BUSCO gene completeness. The main plot is divided into 1,000 size-ordered bins around the circumference with each bin representing 0.1% of the 540,734,767 bp assembly. The distribution of scaffold lengths is shown in dark grey with the plot radius scaled to the longest scaffold present in the assembly (34,005,801 bp, shown in red). Orange and pale-orange arcs show the N50 and N90 scaffold lengths (5,931,830 and 4,666,103 bp), respectively. The pale grey spiral shows the cumulative scaffold count on a log scale with white scale lines showing successive orders of magnitude. The blue and pale-blue area around the outside of the plot shows the distribution of GC, AT and N percentages in the same bins as the inner plot. A summary of complete, fragmented, duplicated and missing BUSCO genes in the lepidoptera_odb10 set is shown in the top right. An interactive version of this figure is available at https://blobtoolkit.genomehubs.org/view/ilLysCori1.1/dataset/CAJNAC01/snail.

The final assembly has a total length of 540.7 Mb in 99 sequence scaffolds with a scaffold N50 of 5.9 Mb ( Table 1). Most (99.92%) of the assembly sequence was assigned to 90 chromosomal-level scaffolds, representing 89 autosomes and the Z sex chromosome. Chromosome-scale scaffolds confirmed by the Hi-C data are named in order of size ( Figure 2– Figure 5; Table 2). While not fully phased, the assembly deposited is of one haplotype. Contigs corresponding to the second haplotype have also been deposited.

**Table 1.** Genome data for *Lysandra coridon*, ilLysCori1.1.

Project accession data		
Assembly identifier	ilLysCori1.1	
Species	*Lysandra coridon*	
Specimen	ilLysCori1	
NCBI taxonomy ID	268709	
BioProject	PRJEB42953	
BioSample ID	SAMEA7523305	
Isolate information	ilLysCori1, male (genome sequencing and RNA sequencing) ilLysCori2, male (Hi-C scaffolding)	
Assembly metrics *		*Benchmark*
Consensus quality (QV)	56.1	*≥ 50*
*k*-mer completeness	99.99%	*≥ 95%*
BUSCO **	C:97.2%[S:96.6%,D:0.6%], F:0.6%,M:2.2%,n:5,286	*C ≥ 95%*
Percentage of assembly mapped to chromosomes	99.92%	*≥ 95%*
Sex chromosomes	Z chromosome	*localised homologous pairs*
Organelles	Mitochondrial genome assembled	*complete single alleles*
Raw data accessions		
PacificBiosciences SEQUEL II	ERR6576318	
10X Genomics Illumina	ERR6054416–ERR6054419	
Hi-C Illumina	ERR6054420, ERR6054421, ERR6054422	
PolyA RNA-Seq Illumina	ERR6286713	
Genome assembly		
Assembly accession	GCA_905220515.1	
*Accession of alternate haplotype*	GCA_905220525.1	
Span (Mb)	540.7	
Number of contigs	338	
Contig N50 length (Mb)	2.0	
Number of scaffolds	99	
Scaffold N50 length (Mb)	5.9	
Longest scaffold (Mb)	34.0	
Genome annotation		
Number of protein-coding genes	13,334	
Average length of coding sequence (bp)	1,435.28	
Average number of exons per transcript	6.99	
Average number of introns per transcript	5.99	
Average intron size (bp)	2,285.81	

^*^Assembly metric benchmarks are adapted from column VGP-2020 of “Table 1: Proposed standards and metrics for defining genome assembly quality” from ( Rhie *et al.*, 2021). ^**^BUSCO scores based on the lepidoptera_odb10 BUSCO set using v5.3.2. C = complete [S = single copy, D = duplicated], F = fragmented, M = missing, n = number of orthologues in comparison. A full set of BUSCO scores is available at https://blobtoolkit.genomehubs.org/view/ilLysCori1.1/dataset/CAJNAC01/busco.

**Figure 3.**
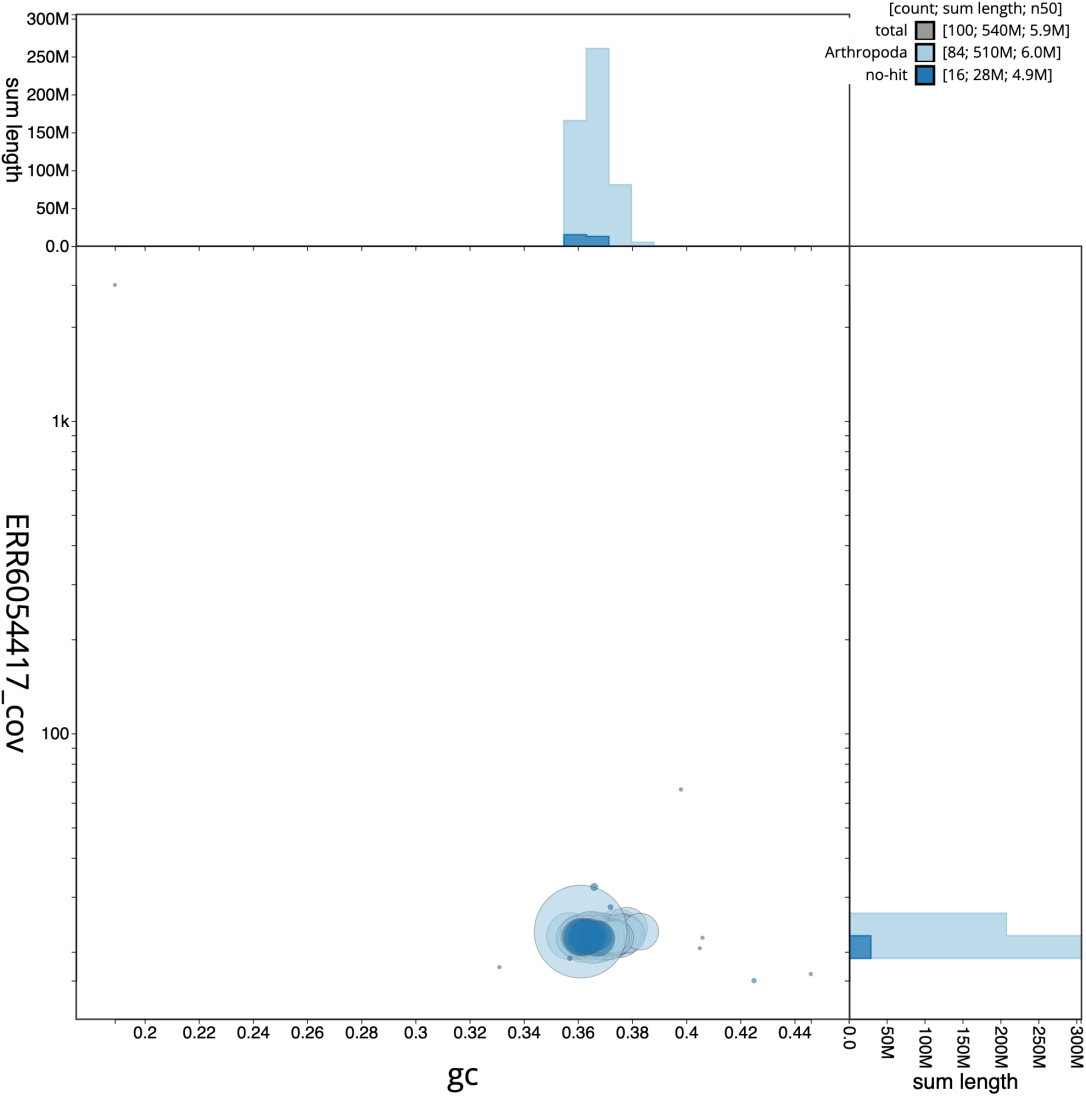
Genome assembly of *Lysandra coridon*, ilLysCori1.1: GC coverage. BlobToolKit GC-coverage plot. Scaffolds are coloured by phylum. Circles are sized in proportion to scaffold length. Histograms show the distribution of scaffold length sum along each axis. An interactive version of this figure is available at https://blobtoolkit.genomehubs.org/view/ilLysCori1.1/dataset/CAJNAC01/blob.

**Figure 4.**
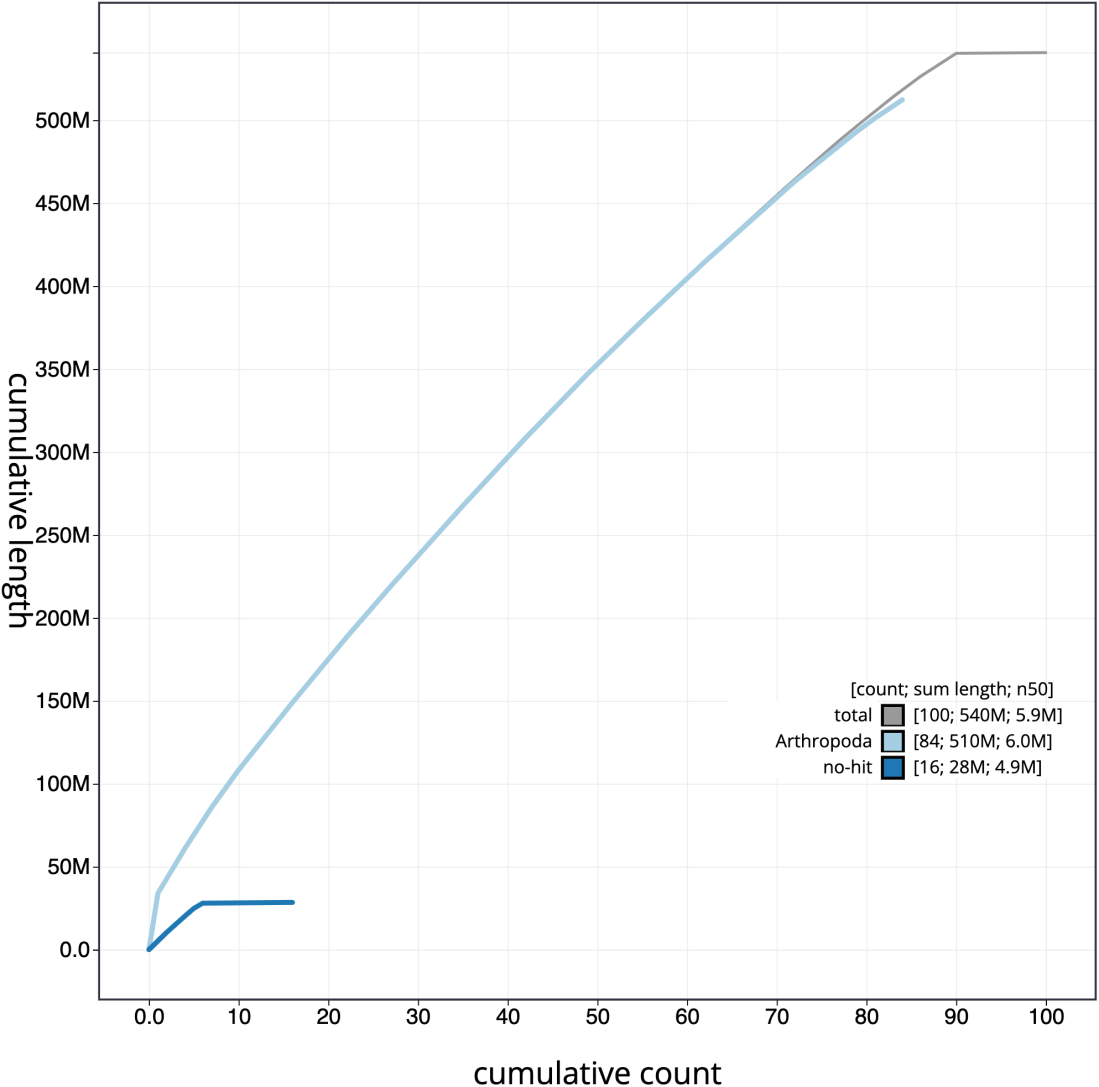
Genome assembly of *Lysandra coridon*, ilLysCori1.1: cumulative sequence. BlobToolKit cumulative sequence plot. The grey line shows cumulative length for all scaffolds. Coloured lines show cumulative lengths of scaffolds assigned to each phylum using the buscogenes taxrule. An interactive version of this figure is available at https://blobtoolkit.genomehubs.org/view/ilLysCori1.1/dataset/CAJNAC01/cumulative.

**Figure 5.**
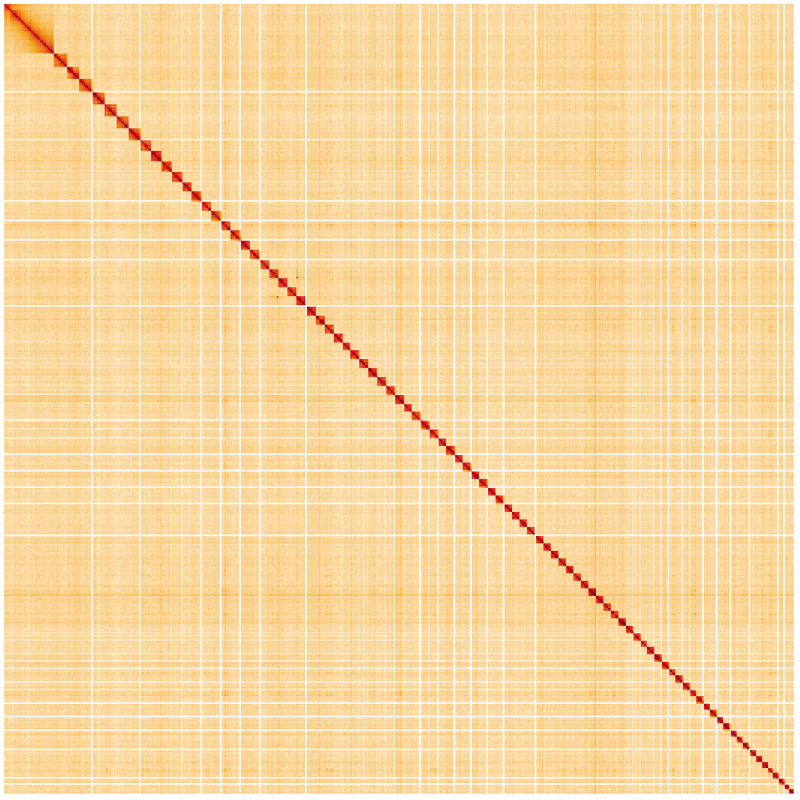
Genome assembly of *Lysandra coridon*, ilLysCori1.1: Hi-C contact map. Hi-C contact map of the ilLysCori1.1 assembly, visualised using HiGlass. Chromosomes are shown in order of size from left to right and top to bottom. An interactive version of this figure may be viewed at https://genome-note-higlass.tol.sanger.ac.uk/l/?d=Vbiq6iyHTQC5rAsnRSQCCQ.

**Table 2.** Chromosomal pseudomolecules in the genome assembly of *Lysandra coridon*, ilLysCori1.

INSDC accession	Chromosome	Size (Mb)	GC%
HG992056.1	1	9.18	36.7
HG992057.1	2	9	37.5
HG992058.1	3	8.73	36.9
HG992059.1	4	8.44	35.7
HG992060.1	5	8.34	36.5
HG992061.1	6	8.14	36.2
HG992062.1	7	7.84	36.1
HG992063.1	8	7.39	36.6
HG992064.1	9	7.22	36.9
HG992065.1	10	6.96	36.9
HG992066.1	11	6.92	37.1
HG992067.1	12	6.75	36
HG992068.1	13	6.7	36.4
HG992069.1	14	6.69	36.8
HG992070.1	15	6.68	37
HG992071.1	16	6.6	37
HG992072.1	17	6.57	36.4
HG992073.1	18	6.56	37.3
HG992074.1	19	6.49	36.5
HG992075.1	20	6.47	37.1
HG992076.1	21	6.45	37.8
HG992077.1	22	6.32	37
HG992078.1	23	6.26	36.3
HG992079.1	24	6.25	36.6
HG992080.1	25	6.21	37.4
HG992081.1	26	6.18	37.3
HG992082.1	27	6.09	36.3
HG992083.1	28	6.07	36.2
HG992084.1	29	6.06	36.2
HG992085.1	30	6.03	36.6
HG992086.1	31	6.03	36.1
HG992087.1	32	6.02	36.5
HG992088.1	33	5.98	36.7
HG992089.1	34	5.95	37.1
HG992090.1	35	5.93	37.6
HG992091.1	36	5.89	36.9
HG992092.1	37	5.87	36.3
HG992093.1	38	5.87	37.4
HG992094.1	39	5.86	36.3
HG992095.1	40	5.77	36.9
HG992096.1	41	5.72	37.7
HG992097.1	42	5.66	36.5
HG992098.1	43	5.65	36.1
HG992099.1	44	5.61	36.8
HG992100.1	45	5.57	36.1
HG992101.1	46	5.54	36.5
HG992102.1	47	5.54	35.9
HG992103.1	48	5.47	37
HG992104.1	49	5.41	36.2
HG992105.1	50	5.37	36.3
HG992106.1	51	5.3	36.8
HG992107.1	52	5.27	37.8
HG992108.1	53	5.24	36.1
HG992109.1	54	5.21	36.4
HG992110.1	55	5.2	37.1
HG992111.1	56	5.15	36.1
HG992112.1	57	5.13	36.4
HG992113.1	58	5.13	37
HG992114.1	59	5.13	38.3
HG992115.1	60	5.12	37.3
HG992116.1	61	5.07	36.6
HG992117.1	62	5.05	36.4
HG992118.1	63	5.03	37.3
HG992119.1	64	4.93	36
HG992120.1	65	4.92	36.5
HG992121.1	66	4.91	37.2
HG992122.1	67	4.91	36.3
HG992123.1	68	4.85	36.7
HG992124.1	69	4.85	37.1
HG992125.1	70	4.83	36.7
HG992126.1	71	4.79	36.4
HG992127.1	72	4.77	36.8
HG992128.1	73	4.76	37
HG992129.1	74	4.69	36.7
HG992130.1	75	4.68	37.4
HG992131.1	76	4.67	36.3
HG992132.1	77	4.46	36
HG992133.1	78	4.45	36.6
HG992134.1	79	4.42	36.3
HG992135.1	80	4.37	36.3
HG992136.1	81	4.34	36.9
HG992137.1	82	4.22	37.1
HG992138.1	83	4.12	36.9
HG992139.1	84	4.03	37.3
HG992140.1	85	3.94	36.3
HG992141.1	86	3.74	36
HG992142.1	87	3.63	36.7
HG992143.1	88	3.49	36.4
HG992144.1	89	3.17	36.7
HG992055.1	Z	34.01	36.1
HG992145.1	MT	0.02	19.2
-	unplaced	0.42	38.1

The estimated Quality Value (QV) of the final assembly is 56.1 with *k*-mer completeness of 99.99%, and the assembly has a BUSCO v5.3.2 completeness of 97.2% (single = 96.6%, duplicated = 0.6%), using the lepidoptera_odb10 reference set ( *n* = 5,286).

### Genome annotation report

The *L. coridon* genome assembly GCA_905220515.1 was generated using the Ensembl genome annotation pipeline ( Table 1; Accession number GCA_905220515.1). The resulting annotation includes 13,334 protein coding genes with an average length of 16,708.51 and an average coding length of 1,435.28, and 2,742 non-protein coding genes. There is an average of 6.99 exons and 5.99 introns per canonical protein coding transcript, with an average intron length of 2,285.81. A total of 5368 gene loci have more than one associated transcript.

## Methods

### Sample acquisition and nucleic acid extraction

Two adult male *L. coridon* specimens were collected on 16 July 2018 using a hand net. The specimen that was used for genome sequencing, ilLysCori1 (voucher no. RO_LC_853), was collected from Săcel, Cluj, Romania (latitude 46.61, longitude 23.46), while the specimen used for Hi-C scaffolding, ilLysCori2 (voucher no. RO_LS_903), was collected from Rimetea, Romania (46.45, 23.58). The collectors were Konrad Lohse and Dominik Laetsch (University of Edinburgh), Alex Hayward (University of Exeter) and Roger Vila (Institut de Biologia Evolutiva, CSIC-UPF). The specimens were identified by Roger Vila and were snap-frozen from live in a dry shipper.

DNA was extracted at the Tree of Life laboratory, Wellcome Sanger Institute (WSI). The ilLysCori1 sample was weighed and dissected on dry ice with tissue set aside for RNA sequencing. Whole organism tissue was disrupted using a Nippi Powermasher fitted with a BioMasher pestle. High molecular weight (HMW) DNA was extracted using the Qiagen MagAttract HMW DNA extraction kit. Low molecular weight DNA was removed from a 20-ng aliquot of extracted DNA using 0.8X AMpure XP purification kit prior to 10X Chromium sequencing; a minimum of 50 ng DNA was submitted for 10X sequencing. HMW DNA was sheared into an average fragment size of 12–20 kb in a Megaruptor 3 system with speed setting 30. Sheared DNA was purified by solid-phase reversible immobilisation using AMPure PB beads with a 1.8X ratio of beads to sample to remove the shorter fragments and concentrate the DNA sample. The concentration of the sheared and purified DNA was assessed using a Nanodrop spectrophotometer and Qubit Fluorometer and Qubit dsDNA High Sensitivity Assay kit. Fragment size distribution was evaluated by running the sample on the FemtoPulse system.

RNA was extracted from ilLysCori1 in the Tree of Life Laboratory at the WSI using TRIzol, according to the manufacturer’s instructions. RNA was then eluted in 50 μL RNAse-free water and its concentration assessed using a Nanodrop spectrophotometer and Qubit Fluorometer using the Qubit RNA Broad-Range (BR) Assay kit. Analysis of the integrity of the RNA was done using Agilent RNA 6000 Pico Kit and Eukaryotic Total RNA assay.

### Sequencing

Pacific Biosciences HiFi circular consensus and 10X Genomics read cloud DNA sequencing libraries were constructed according to the manufacturers’ instructions. Poly(A) RNA-Seq libraries were constructed using the NEB Ultra II RNA Library Prep kit. DNA and RNA sequencing was performed by the Scientific Operations core at the WSI on Pacific Biosciences SEQUEL II (HiFi), Illumina HiSeq 4000 Q (RNA-Seq) and HiSeq X Ten (10X) instruments. Hi-C data were also generated from whole organism tissue of ilLysCori2 using the Arima v2 kit and sequenced on the HiSeq X Ten instrument.

### Genome assembly, curation and evaluation

Assembly was carried out with Hifiasm ( Cheng *et al.*, 2021) and haplotypic duplication was identified and removed with purge_dups ( Guan *et al.*, 2020). One round of polishing was performed by aligning 10X Genomics read data to the assembly with Long Ranger ALIGN, calling variants with FreeBayes ( Garrison & Marth, 2012). The assembly was then scaffolded with Hi-C data ( Rao *et al.*, 2014) using SALSA2 ( Ghurye *et al.*, 2019). The assembly was checked for contamination and corrected using the gEVAL system ( Chow *et al.*, 2016) as described previously ( Howe *et al.*, 2021). Manual curation was performed using gEVAL, HiGlass ( Kerpedjiev *et al.*, 2018) and Pretext ( Harry, 2022). The mitochondrial genome was assembled using MitoHiFi ( Uliano-Silva *et al.*, 2022), which performed annotation using MitoFinder ( Allio *et al.*, 2020). To evaluate the assembly, MerquryFK was used to estimate consensus quality (QV) scores and *k*-mer completeness ( Rhie *et al.*, 2020). The genome was analysed and BUSCO scores ( Manni *et al.*, 2021; Simão *et al.*, 2015) were calculated within the BlobToolKit environment ( Challis *et al.*, 2020). Table 3 contains a list of software tool versions and sources.

**Table 3.** Software tools: versions and sources.

Software tool	Version	Source
BlobToolKit	3.5.2	https://github.com/blobtoolkit/blobtoolkit
BUSCO	5.3.2	https://gitlab.com/ezlab/busco
FreeBayes	1.3.1-17-gaa2ace8	https://github.com/freebayes/freebayes
gEVAL	N/A	https://geval.org.uk/
Hifiasm	0.12	https://github.com/chhylp123/hifiasm
HiGlass	1.11.6	https://github.com/higlass/higlass
Long Ranger ALIGN	2.2.2	https://support.10xgenomics.com/genome-exome/ software/pipelines/latest/advanced/other-pipelines
Merqury	MerquryFK	https://github.com/thegenemyers/MERQURY.FK
MitoHiFi	2	https://github.com/marcelauliano/MitoHiFi
PretextView	0.2	https://github.com/wtsi-hpag/PretextView
purge_dups	1.2.3	https://github.com/dfguan/purge_dups
SALSA	2.2	https://github.com/salsa-rs/salsa

### Genome annotation

The Ensembl gene annotation system ( Aken *et al.*, 2016) was used to generate annotation for the *L. coridon* assembly (GCA_905220515.1). Annotation was created primarily through alignment of transcriptomic data to the genome, with gap filling via protein to-genome alignments of a select set of proteins from UniProt ( UniProt Consortium, 2019).

### Ethics/compliance issues

The materials that have contributed to this genome note have been supplied by a Darwin Tree of Life Partner. The submission of materials by a Darwin Tree of Life Partner is subject to the Darwin Tree of Life Project Sampling Code of Practice. By agreeing with and signing up to the Sampling Code of Practice, the Darwin Tree of Life Partner agrees they will meet the legal and ethical requirements and standards set out within this document in respect of all samples acquired for, and supplied to, the Darwin Tree of Life Project. Each transfer of samples is further undertaken according to a Research Collaboration Agreement or Material Transfer Agreement entered into by the Darwin Tree of Life Partner, Genome Research Limited (operating as the Wellcome Sanger Institute), and in some circumstances other Darwin Tree of Life collaborators.

## Data Availability

European Nucleotide Archive: *Lysandra coridon* (chalkhill blue). Accession number PRJEB42953; https://identifiers.org/ena.embl/PRJEB42953 ( Wellcome Sanger Institute, 2021) The genome sequence is released openly for reuse. The *Lysandra coridon* genome sequencing initiative is part of the Darwin Tree of Life (DToL) project. All raw sequence data and the assembly have been deposited in INSDC databases. Raw data and assembly accession identifiers are reported in Table 1.

## Author information

Members of the Wellcome Sanger Institute Tree of Life programme are listed here: https://doi.org/10.5281/zenodo.4783585.

Members of Wellcome Sanger Institute Scientific Operations: DNA Pipelines collective are listed here: https://doi.org/10.5281/zenodo.4790455.

Members of the Tree of Life Core Informatics collective are listed here: https://doi.org/10.5281/zenodo.5013541.

Members of the Darwin Tree of Life Consortium are listed here: https://doi.org/10.5281/zenodo.4783558.
