# Supplementary material for: The genome sequence of the Chalkhill Blue, Lysandra coridon (Poda, 1761)
Source: Wellcome Open Res. 2023 Apr 12;8:162. [Version 1] doi: 10.12688/wellcomeopenres.19253.1 (PMC11103778; doi:10.12688/wellcomeopenres.19253.1)
Supplement: Peer review history

# Reviewer report: Reviewer response for version 1

**DOI:** 10.21956/wellcomeopenres.21336.r72958

**Published:** 2024-05-17

**Authors:** Duncan Sivell

**Affiliations:** 1 Natural History Museum, Cromwell Road, London, UK

Although the authors successfully present a genome for *Lysandra corison* they do not explain why they have sequenced a specimen that is genetically different to the target population. Romanian specimens have been used for genome sequencing even though the DToL project is focused on sequencing British material. I assume there may have been extenuating circumstances why this has happened (e.g. COVID lockdowns?) but considering *Lysandra coridon* is not a difficult species to find on chalk in the south of England it does seem strange that UK material has not been processed. The authors note that populations in western and eastern Europe belong to different genetic lineages, which would seem to support the need to sequence UK butterflies over Romanian ones.

 I have no issues with the text or methods in this paper, but the choice of specimens does not fit with the rational of the project.

Are sufficient details of methods and materials provided to allow replication by others?

Yes

Is the rationale for creating the dataset(s) clearly described?

Partly

Are the datasets clearly presented in a useable and accessible format?

Yes

Are the protocols appropriate and is the work technically sound?

Yes

Reviewer Expertise:

Entomology and ecology.

I confirm that I have read this submission and believe that I have an appropriate level of expertise to confirm that it is of an acceptable scientific standard, however I have significant reservations, as outlined above.

# Reviewer report: Reviewer response for version 1

**DOI:** 10.21956/wellcomeopenres.21336.r79623

**Published:** 2024-05-16

**Authors:** Yaohui Wang

**Affiliations:** 1 Anhui Agricultural University, Shanghai Institute of Plant Physiology and Ecology, Chinese Academy of Sciences, Shanghai, China

**Comments for author**

 The authors present their work on *Lysandra coridon*, a species of butterfly that typically inhabits calcareous grasslands through-out Europe. The importance of the subject is very high. It is overall a nice piece of work in Lycaenidae genome assembly and Gene annotation, due to both major ecological importance and the closeness of the species to the butterfly community. I think this will be and example for the importance of gene content change analysis.

 In summary it is an important genome resource that is critically needed. It is pretty short and well written.

**Major issue**

 As they are currently written, there are also some methods that need to be clarified. the methods do not provide sufficient detail for other scientists who wish to reproduce them. I would suggest that the authors include a greatly expanded methods section in their Supplementary Material, which describes parameter settings for each software used in their data analysis.

Are sufficient details of methods and materials provided to allow replication by others?

Partly

Is the rationale for creating the dataset(s) clearly described?

Yes

Are the datasets clearly presented in a useable and accessible format?

Yes

Are the protocols appropriate and is the work technically sound?

Yes

Reviewer Expertise:

Omics analysis, Genetic regulation, Gene editing

I confirm that I have read this submission and believe that I have an appropriate level of expertise to confirm that it is of an acceptable scientific standard.

# Reviewer report: Reviewer response for version 1

**DOI:** 10.21956/wellcomeopenres.21336.r79628

**Published:** 2024-05-06

**Authors:** Huateng Huang, Kuo Sun

**Affiliations:** 1 Shaanxi Normal University, Xi’an, China; 2 Shaanxi Institute of zoology, Xi'an, Shaanxi, China

This article introduces a newly assembled genome for the Chalkhill Blue butterfly, *Lysandra coridon* (Poda, 1761) (Lepidoptera, Lycaenidae). Through the integration of data from Pacific Biosciences, Illumina HiSeq 10X, and Hi-C technologies, the authors have constructed a high-quality assembly. This assembly consists of 90 chromosomal pseudomolecules, encompassing both the Z sex chromosome and the mitochondrial genome. The methods utilized are thoroughly described, and the results are presented with clarity. Undoubtedly, this assembly serves as a valuable resource for future investigations.

 I just have one minor suggestion:

 The authors could provide additional context regarding the significance of whole-genome sequencing for the conservation of the Chalkhill Blue butterfly in the introduction section.

Are sufficient details of methods and materials provided to allow replication by others?

Yes

Is the rationale for creating the dataset(s) clearly described?

Yes

Are the datasets clearly presented in a useable and accessible format?

Yes

Are the protocols appropriate and is the work technically sound?

Yes

Reviewer Expertise:

evolutionary biology

We confirm that we have read this submission and believe that we have an appropriate level of expertise to confirm that it is of an acceptable scientific standard.

## References

[1] : The preimaginal epidermal organs of Lycaena tityrus (Poda, 1761) and Polyommatus coridon (Poda, 1761) (Lepidoptera: Lycaenidae) - a comparison. *Nota Lepidopterologica 11(2):100-116* .1988;
